# Evolutionary paths to macrolide resistance in a *Neisseria* commensal converge on ribosomal genes through short sequence duplications

**DOI:** 10.1371/journal.pone.0262370

**Published:** 2022-01-13

**Authors:** Jordan C. Raisman, Michael A. Fiore, Lucille Tomin, Joseph K. O. Adjei, Virginia X. Aswad, Jonathan Chu, Christina J. Domondon, Ben A. Donahue, Claudia A. Masciotti, Connor G. McGrath, Jo Melita, Paul A. Podbielski, Madelyn R. Schreiner, Lauren J. Trumpore, Peter C. Wengert, Emalee A. Wrightstone, André O. Hudson, Crista B. Wadsworth

**Affiliations:** Rochester Institute of Technology, Thomas H. Gosnell School of Life Sciences, Rochester, NY, United States of America; University of Iceland, ICELAND

## Abstract

*Neisseria* commensals are an indisputable source of resistance for their pathogenic relatives. However, the evolutionary paths commensal species take to reduced susceptibility in this genus have been relatively underexplored. Here, we leverage *in vitro* selection as a powerful screen to identify the genetic adaptations that produce azithromycin resistance (≥ 2 μg/mL) in the *Neisseria* commensal, *N*. *elongata*. Across multiple lineages (n = 7/16), we find mutations that reduce susceptibility to azithromycin converge on the locus encoding the 50S ribosomal L34 protein (*rpmH*) and the intergenic region proximal to the 30S ribosomal S3 protein (*rpsC)* through short tandem duplication events. Interestingly, one of the laboratory evolved mutations in *rpmH* is identical (7LKRTYQ12), and two nearly identical, to those recently reported to contribute to high-level azithromycin resistance in *N*. *gonorrhoeae*. Transformations into the ancestral *N*. *elongata* lineage confirmed the causality of both *rpmH* and *rpsC* mutations. Though most lineages inheriting duplications suffered *in vitro* fitness costs, one variant showed no growth defect, suggesting the possibility that it may be sustained in natural populations. Ultimately, studies like this will be critical for predicting commensal alleles that could rapidly disseminate into pathogen populations via allelic exchange across recombinogenic microbial genera.

## Introduction

Commensal bacterial populations have been increasingly recognized for their importance as sources of adaptive genetic variation for pathogens through horizontal gene transfer (HGT) [1–4], however non-pathogenic species are less frequently characterized as they are less of a danger to public health [5]. The threat of rapid evolution as a result of DNA donation is especially amplified in highly recombinogenic genera, such as the *Neisseria*. Members of this genus readily donate DNA to one another through pilus-mediated *Neisseria*-specific DNA uptake and homologous recombination [6–8]. Observations of genetic mosaicism, whereby loci within a particular lineage have been acquired from another species, are common [9–16] and occur genome-wide [17,18]. This promiscuous allelic exchange has been documented to have facilitated rapid adaptive evolution of important phenotypic characteristics such as antimicrobial resistance [6,8–10] and body-site colonization niche shifts [16]. Overall, recently incorporated mosaic sequences show signatures consistent with positive selection [17], suggesting intragenus recombination is an important source of beneficial genetic variation.

The Gram-negative genus *Neisseria* is comprised of several species that typically colonize the mucosa of humans and animals. Most human-associated *Neisseria* inhabit the naso- and oropharynx, and are carried harmlessly as commensals in 10–15% of healthy human adults and children [19,20]. However, two species within the genus are important human pathogens. *N*. *meningitidis* (Nmen) can cause meningococcal disease, which can result in septicemia and meningitis, though pharyngeal carriage is most typically asymptomatic and rarely leads to severe invasive disease [19,20]. *N*. *gonorrhoeae* (Ngo) is an obligate pathogen, which in addition to the naso- and oropharyngeal mucosa, also colonizes the urogenital tract and rectum and causes the sexually transmitted infection gonorrhea [21]. In recent years multiple studies have documented that *Neisseria* commensals serve as important reservoirs of adaptive genetic variation for Ngo and Nmen through HGT [11–18]. As pathogens and commensals share common colonization sites in the naso- and oropharynx [14–16], close proximately and contact in these sites likely facilitates the high rates of recombination observed between species. However, most of the non-pathogenic *Neisseria* have been infrequently characterized as they rarely cause life-threatening disease, except in immunocompromised individuals [22,23].

Horizontal transfer and subsequent spread of commensal *Neisseria* resistance mechanisms has historically played a large role in rendering antibiotic therapies ineffective in Ngo and Nmen. In a U.S. gonococcal population dataset, over 11% of reduced susceptibility to azithromycin was acquired through inheritance of commensal *multiple transferable efflux pump* (*mtr*) alleles [13–15]. Additionally, the majority of resistance to third-generation cephalosporins in gonococci is derived through mosaic *penicillin-binding protein 2* (*penA*) alleles obtained from close commensal relatives [11,15]. In Nmen, resistance to quinolones has been shown to be directly acquired from commensals though transfer of mutations in the quinolone resistance-determining region (QRDR) of the *gyrA* gene (encoding the subunit A of DNA gyrase) [24,25]. As reduced drug susceptibility in commensal *Neisseria* populations has been shown to be directly selected for after antibiotic usage [26] these species will always present a persistent threat for resistance donation. Characterization of the resistance genotypes and phenotypes in panels of commensal *Neisseria* (such as [27–29]) will aid in prospective surveillance for novel resistance that may be rapidly acquired by Ngo and Nmen, though the utility of these approaches is ultimately limited by extensive under sampling of natural commensal populations.

Experimental evolution can be leveraged as an alternative approach for nominating the mechanisms that underly antibiotic resistance across the commensal *Neisseria*, and thus may be at risk of HGT to pathogenic *Neisseria*. Laboratory evolution experiments reveal the spontaneous mutations caused by DNA replication and repair errors which increase mean fitness in new selective environments [30], and are ideal to elucidate the mechanisms underlying novel adaptations in bacteria due to their short generation times. *In vitro* selection has previously been used successfully to nominate the mutations underlying ceftriaxone and azithromycin reduced susceptibility in Ngo [31,32], and thus is a promising tool for the discovery of undescribed resistance mechanisms in *Neisseria* commensals.

In this study we use antibiotic-mediated selection to investigate the potential for a *Neisseria* commensal (*N*. *elongata*, Nel) to evolve resistance to the macrolide antibiotic azithromycin. Azithromycin binds to the 23S rRNA of the bacterial 50S ribosomal subunit, and was recommended as a first-line therapy in combination with ceftriaxone, for the treatment of uncomplicated cases of gonorrhea in the United States and the United Kingdom until 2020 and 2019 respectively [33,34]. After selection, we characterized the evolutionary response of Nel to azithromycin and considered the number of adaptive solutions across replicate lineages using whole genomic sequencing. We finally confirmed the causality of resistance mutations through transformation, and assessed the fitness costs of derived mutations in the ancestral genomic background.

## Results

### Evolutionary trajectories to macrolide resistance in *N*. *elongata*

*N*. *elongata* AR Bank #0945 was selected to explore the evolutionary paths to azithromycin resistance in a *Neisseria* commensal. *N*. *elongata* AR Bank #0945 has been tested for its minimum inhibitory concentration (MIC) to azithromycin (0.38 μg/mL) and sequenced (SAMN15454046) by our group previously [28]. In this study, a draft genome was assembled as an ancestral reference for all derived lineages. The assembly contained 2,572,594 bps, and 2,509 annotated genes (JAFEUH000000000).

**Fig 1:**
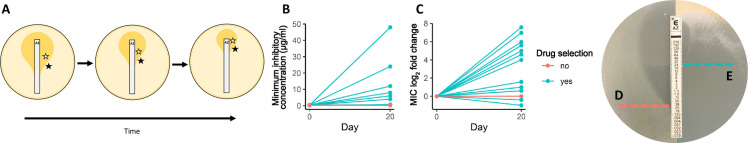
*In vitro* evolution under azithromycin selection produces high-level resistance in multiple replicate lineages of the *Neisseria* commensal, *N*. *elongata*. (A) Parallel cultures of *N*. *elongata* AR Bank #0945 were passaged across an Etest-generated concentration gradient of azithromycin, selecting any cell growth in the zones of inhibition (white stars) and a 1 cm band at the edge of the bacterial lawn (black stars) for 20 days (n = 16 replicates). (B,C) In 7 of the 16 lineages, reduced susceptibility as defined by the CLSI cutoff of ≥ 2 μg/mL emerged. In all cases, MICs for evolved lineages increased from the ancestral *N*. *elongata* AR Bank #0945 strain value of 0.38 μg/mL (D), with strain LT1 displayed (E).

Selective pressure was applied to sixteen replicates of AR Bank #0945 by creating a concentration gradient of azithromycin on standard growth media via application of Etest strips (Fig 1A). Cells were selected for passaging by sweeping the entire zone of inhibition (ZOI) and a 1 cm band in the bacterial lawn adjacent to the ZOI (Fig 1A). After 20 days, or approximately 480 generations, the average MIC value for all evolved cell populations increased to 7.6 μg/mL, which was significantly higher compared to day one values (*W* = 98.5, *P* = 0.0004), and ranged from 0.19 to 48 μg/mL (Fig 1B and 1C; S1 Fig). Control populations with no drug selection (n = 4), showed no increase in MIC compared to the ancestral stock (Fig 1B and 1C; S1 Fig). To mitigate the possibility of heterogenous cultures at the termination of the experiment, a single colony was selected from each evolved and control population for further MIC testing and genomic sequencing. MIC values for drug-selected single colony picks tended to be higher than those recorded for population values, and ranged from 0.5 to 64 μg/mL, with an average value of 14.4 μg/mL (Table 1).

**Table 1:** Azithromycin MICs and derivation data for the strains used in this study.

Strain	Post-selection MIC (μg//ml) d	Recipient strain	Donor Strain
**Selected strains a**			
AM1	24	-	-
CBW1	1	-	-
CBW2	1	-	-
CBW3	1	-	-
CBW4	64	-	-
CBW5	0.5	-	-
CBW6	64	-	-
EW1	0.5	-	-
JA1	8	-	-
JC1	0.5	-	-
JM1	0.75	-	-
JR1	1	-	-
LJT1	16	-	-
LT1	24	-	-
MRS1	24	-	-
PAP1	0.75	-	-
**Control strains b**			
ND1	0.38	-	-
ND2	0.38	-	-
ND3	0.38	-	-
ND4	0.38	-	-
**Transformants**			
T-CBW4-1	48	AR-0945	CBW4
T-CBW4-2	48	AR-0945	CBW4
T-CBW4-3	48	AR-0945	CBW4
T-CBW6-1	64	AR-0945	CBW6
T-CBW6-2	64	AR-0945	CBW6
T-CBW6-3	64	AR-0945	CBW6
T-LT1-1	24	AR-0945	LT1
T-LT1-2	24	AR-0945	LT1
T-LT1-3	24	AR-0945	LT1
T-MRS1-1	24	AR-0945	MRS1
T-MRS1-2	24	AR-0945	MRS1
T-MRS1-3	24	AR-0945	MRS1
T-JA1-1	8	AR-0945	JA1
T-JA1-2	8	AR-0945	JA1
T-JA1-3	8	AR-0945	JA1
T-LJT1-1	12	AR-0945	LJT1
T-LJT1-2	12	AR-0945	LJT1
T-LJT1-3	12	AR-0945	LJT1
T-AM1-1	24	AR-0945	AM1
T-AM1-2	24	AR-0945	AM1
T-AM1-3	12	AR-0945	AM1

a. Selection on an azithromycin concentration gradient

b. Strains were passaged for 20 days on GCB-K agar plates with no azithromycin selection

c. Reported MICs are the mode of 3 independent tests.

Evolved cell lines were sequenced and aligned to the *N*. *elongata* AR Bank #0945 draft assembly to nominate derived polymorphisms. Mutations that were shared with control strains, or those shared with ancestral reads mapped back to the reference assembly, were not further considered. In total, 37 derived mutations were identified across all sequenced strains (Table 2). The most frequently observed mutations were found in the glucokinase encoded by *glk* (n = 13 lineages), followed by those in *rpmH* encoding the 50S ribosomal protein L34 (n = 4), and mutations in the intergenic region proximal to *rpsC* encoding the 30S ribosomal S3 protein (n = 3). Unique mutations observed within annotated genes, were present in the coding domains for: the capsular polysaccharide phosphotransferase (encoded by *cps12A*), isocitrate lyase (*aceA*), RNA 2’-phosphotransferase (*kptA*), the di-/tripeptide transporter (*dptT*), and the bifunctional (p)ppGpp synthase/hydrolase (*spoT*). All strains with azithromycin MIC values ≥ 2 μg/mL (the Clinical & Laboratory Standards Institute (CLSI) reduced susceptibility breakpoint for *N*. *gonorrhoeae* [35]) were associated inheritance of mutations in either *rpmH* or the intergenic region proximal to *rpsC*.

**Table 2:** Derived SNPs in evolved lineages annotated in reference to the draft AR-0945 assembly (JAFEUH000000000).

Strain	Node	Position	Ancestral Variant	Derived Allele	Gene	Product
AM1	NODE 1	283041	G	GGTACGTTTGCGTTTGGTTACGGAA	*rpmH*	50S ribosomal protein L34
AM1	NODE 23	26465	C	T	*glk*	Glucokinase
CBW1	NODE 23	26537	C	A	*glk*	Glucokinase
CBW1	NODE 3	56344	G	A	*-*	Hypothetical protein
CBW1	NODE 20	6687	A	insertionq	*-*	Hypothetical protein
CBW1	NODE 33	1191	G	insertionq	*-*	
CBW1	NODE 88	352	T	TTGCGCCA	*-*	Hypothetical protein
CBW2	NODE 23	26470	T	A	*glk*	Glucokinase
CBW2	NODE 1	58804	CA	C	*cps12A*	Capsular polysaccharide phosphotransferase
CBW2	NODE 88	352	T	TTGCGCCA	*-*	Hypothetical protein
CBW3	NODE 23	26378	G	A	*glk*	Glucokinase
CBW4	NODE 23	26378	G	A	*glk*	Glucokinase
CBW4	NODE 1	283066	G	GGTTGATAAGTGCGTTTCATGATAT	*rpmH*	50S ribosomal protein L34
CBW5	NODE 23	27739	A	AACGTGTTCATTGTCT	*kptA*	RNA 2’-phosphotransferase
CBW5	NODE 86	404	A	ATGCCTTCTTCCTCACAC	*-*	
CBW6	NODE 23	26378	G	A	*glk*	Glucokinase
CBW6	NODE 4	23104	C	CTTAGCTCGAGCCTGAAAGCGTTTT	Intergenic: *rpsC*, *rplB*	
CBW6	NODE 88	352	T	TTGCGCCA	*-*	
EW1	NODE 1	99142	T	C	Proximal: *porB*	
JA1	NODE 23	26920	G	A	*glk*	Glucokinase
JA1	NODE 84	429	A	AATAAGACGGTGTTGTCGGCAG	*-*	
JA1	NODE 1	283066	G	GGTTGATAAGTGCGTTTCA	*rpmH*	
JC1	NODE 23	26623	G	A	*glk*	Glucokinase
JC1	NODE 20	6968	TG	T	*dtpT*	Di-/tripeptide transporter
JR1	NODE 23	26029	A	G	*glk*	Glucokinase
JR1	NODE 88	352	T	TTGCGCCA	*-*	Hypothetical protein
LJT1	NODE 23	26182	CG	C	*glk*	Glucokinase
LJT1	NODE 4	23096	C	CCACGACCCTTAGCTCGAG	Intergenic: *rpsC*, *rplB*	
LJT1	NODE 88	352	T	TTGCGCCA	*-*	Hypothetical protein
LT1	NODE 23	26393	G	A	*glk*	Glucokinase
LT1	NODE 17	20583	A	G	*aceA*	Isocitrate lyase
LT1	NODE 1	283041	G	GGTACGTTTGCGTTTGGTTACGGAA	*rpmH*	
MRS1	NODE 4	23055	C	CCGTCACAGCAATATG	Intergenic: *rpsC*, *rplB*	
MRS1	NODE 23	26551	G	T	*glk*	Glucokinase
PAP1	NODE 21	14412	G	A	*spoT*	Bifunctional (p)ppGpp synthase/hydrolase SpoT
PAP1	NODE 23	26575	GC	G	*glk*	Glucokinase
PAP1	NODE 88	352	T	TTGCGCCA	*-*	Hypothetical protein

q. insertions over 100 bp are not fully reported.

### Confirmation of the causality of high-level resistance encoding mutations

**Fig 2:**
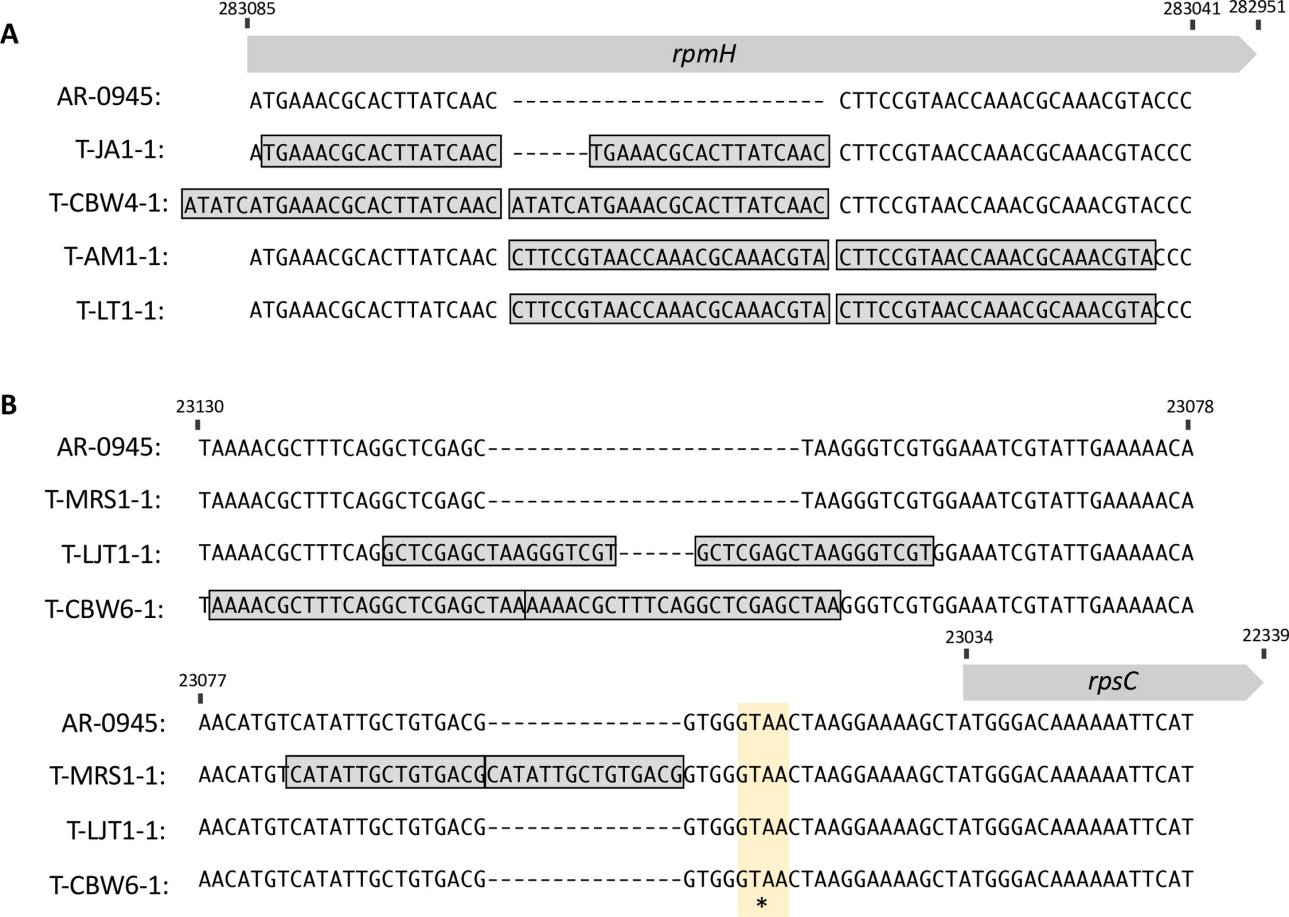
Repeated emergence of mutations within and proximal to genes encoding ribosomal proteins are causal to high-level azithromycin resistance. (A) Whole genomic sequencing revealed that four strains evolved duplications in *rpmH*, the gene encoding the 50S ribosomal protein L34. Transformants (T-AM1, T-CBW4, T-JA1, and T-LT1) generated from these lineages were Sanger sequenced to confirm the location and identity of nominated derived insertional polymorphisms, data shown. (B) The remaining mutations underlying high-level resistance (n = 3) were located proximally to *rpsC*, which encodes the 30S ribosomal protein S3, and were also duplication events. Sanger sequences for the transformants (T-CBW6, T-LJT1, and T-MRS1) are displayed. Reference positions in Node 1 and Node 4 of the AR-0945 draft assembly are displayed for *rpmH* and *rpsC* respectively. The highlighted GTAA region is duplicated in the 3 of 18 Nel genomes deposited on NCBI (S1 Table).

**Fig 3:**
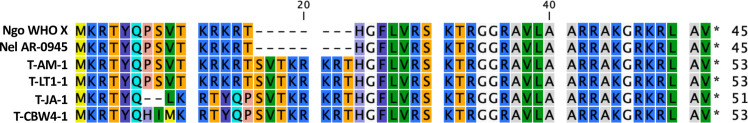
At both the nucleotide and amino acid level Nel AR-0945 RpmH is identical to the Ngo WHO F reference (LT591897.1) sequence. Evolved in-frame sequence duplications in *Nel* lineages introduce 8SVTKRKRT15, 7LKRTYQ12, and 8HIMKRTYQ15 insertions, which are identical to (7LKRTYQ12) and nearly identical to those recently discovered to confer high-level macrolide resistance in Ngo [32].

To assess the causality of the mutations encoding macrolide resistance in *N*. *elongata* AR Bank #0945, the ancestral stock was transformed with genomic DNA from evolved cell lines with MIC values ≥ 2 μg/mL (n = 7). Genomic DNA from evolved lineages successfully transformed the ancestral stock in all cases. To identify causal loci, three colonies from each transformation were selected to characterize the polymorphisms which had been inherited from donor strains that were not present in the AR Bank #0945 ancestral recipient. The only region inherited across replicate transformant colony picks contained either mutations in *rpmH* (DNA donated from AM1, CBW4, JA1, or LT1), or the intergenic region near *rpsC* (DNA donated from CBW6, LJT1, or MRS1) (Table 2). Sanger sequencing confirmed the identity and presence of these mutations (Fig 2), and translation of *rpmH* duplications at the amino acid level indicated the in-frame insertions: 8SVTKRKRT15, 7LKRTYQ12, and 8HIMKRTYQ15 (Fig 3).

In most cases recovered transformants had azithromycin MICs that perfectly mirrored the donor strain phenotypes. However, all three transformants with CBW4 as a donor consistently had phenotypes of 48 μg/mL, below that of the donor strain phenotype of 64 μg/mL (Table 1). Additionally, LJT1 transformants had MIC values of 12 μg/mL, also below that of the donor strain phenotype (Table 1). Finally, one of the three AM1 transformants (T-AM1-3) had a lower MIC (12 μg/mL) than the other transformants and the AM1 donor strain (24 μg/mL).

We also assessed if any *rpmH* or *rpsC* duplication mutations were present in the Nel genomes reported on NCBI (n = 18). We found no duplications within *rpmH*, however there were 3 genomes with a GTAA mutations present 17 bps upstream of the *rpsC* start site (Fig 2; S1 Table).

### Most novel ribosomal variants reduce *in vitro* fitness

**Fig 4:**
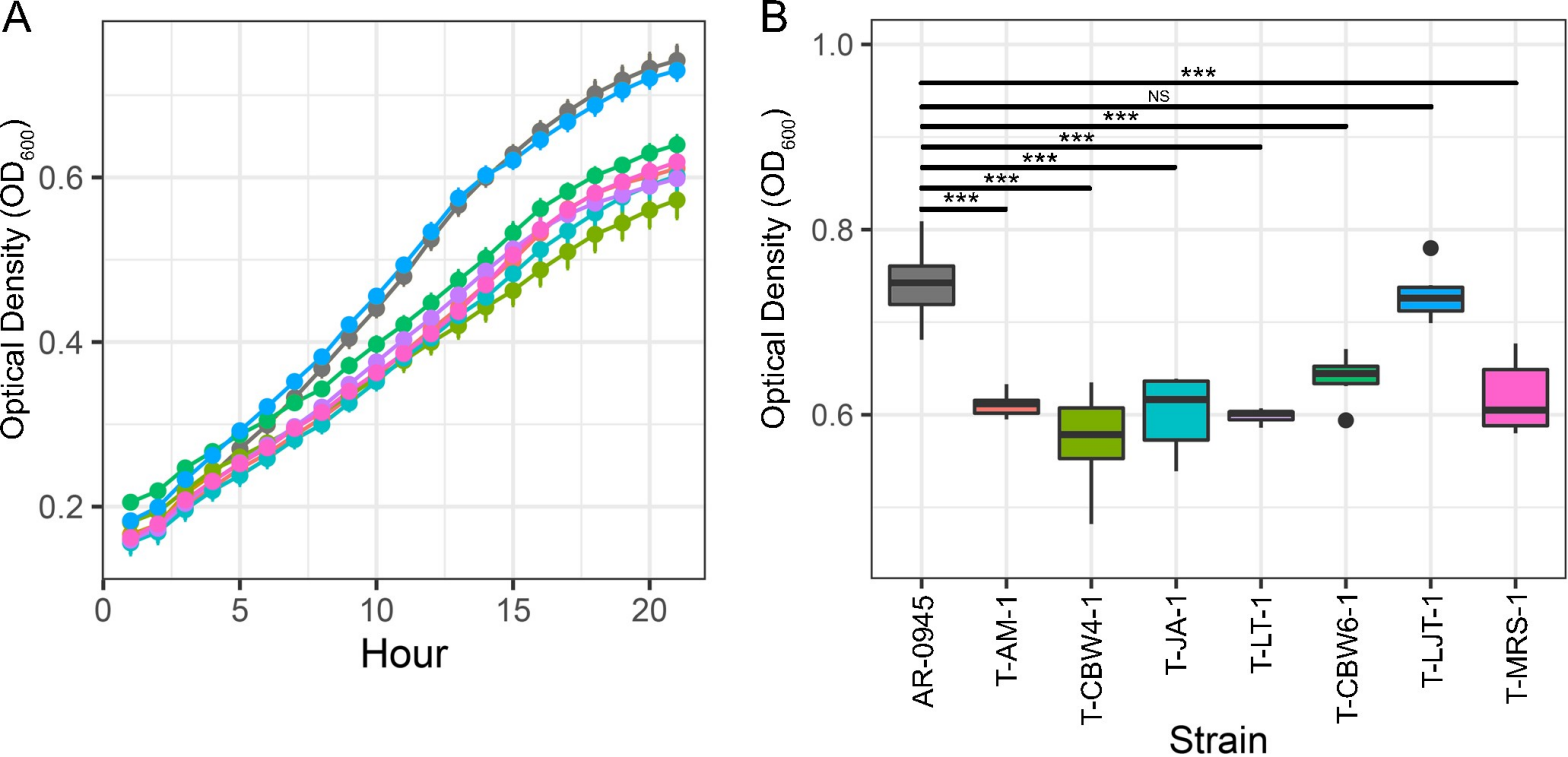
Impact of novel alleles in evolved cell lineages on growth rate compared to the ancestral AR-0945 strain. (A) Growth curves in GCP broth supplemented 1% Kelloggs solution were obtained by monitoring OD_600_ over 21 hours (n = 6 replicates per strain). (B) Growth was significantly reduced in all strains compared to the ancestral at 21 hours, except for strain T-LJT-1. Significance between strains was determined using a one-way ANOVA followed post-hoc by Tukey’s HSD where ***  = *p* < 0.001 and NS = not significant.

To evaluate the fitness costs of azithromycin resistance-conferring mutations, the optical densities of transformant cells lines were compared to the ancestral *N*. *elongata* AR Bank #0945 strain over a 21-hour period (Fig 4A). At hour 21, OD_600_ values for AR-0945 replicates ranged from 0.68 to 0.81 (n = 6); and six of the seven transformants had significantly lower optical densities (Fig 4B; Tukey’s HSD, p < 0.001). OD_600_ values for T-LJT-1 however ranged from 0.70–0.78 (n = 6), which were not significantly different compared to the ancestral strain (Fig 4B; Tukey’s HSD, p < 0.99).

## Discussion

Multiple studies have demonstrated that commensal *Neisseria* serve as reservoirs of resistance for their pathogenic relatives [11,13–15,24,25]. However a comprehensive evaluation of the resistance alleles commensals can harbor, though underway [27–29], and their likelihood of transfer to pathogenic relatives is still in its infancy. Here, we use experimental evolution to screen for the mutations that impart azithromycin resistance in the *Neisseria* commensal Nel.

Overall, our results support constrained evolutionary trajectories to high-level macrolide resistance in Nel. Though azithromycin resistance in most Nmen populations is rare [e.g., 36,37] except in a novel urogenital-colonizing clade [38], a diversity of resistance mutations have been reported in Ngo including: alterations in the 23S rRNA azithromycin binding sites (C2611T and A2059G) [39,40], a G70D mutation in the RplD 50S ribosomal protein L4 [41], *rplV* tandem duplications [20], variants of the rRNA methylase genes *ermC* and *ermB* [42], and mutations that enhance the expression of Mtr or increase the binding efficiency of MtrD with its substrates [43–45]. Of the aforementioned mutations in Ngo however high-level resistance is most frequently imparted by ribosomal mutations [15,41]. Similarly, for all cases of high-level resistance emergence in Nel drug-selected lineages within this study, causal mutations converged on the gene encoding the 50S ribosomal L34 protein (*rpmH*) or the intergenic region proximal to the 30S ribosomal S3 protein (*rpsC)*. Interestingly, in all cases Nel resistance-conferring mutations evolved through short tandem duplication events.

The evolved L34 mutations uncovered within this study in Nel are identical or nearly identical to those recently reported to have emerged in an azithromycin-selection study in Ngo. The L34 duplications 7PSVTKRKR14, 7PSVTNTYQP14, and 7LKRTYQ12 were found to contribute to high levels of resistance to azithromycin in multiple Ngo strains [32]; and occurred in similar or the same locations as the Nel duplications 8SVTKRKRT15, 8HIMKRTYQ15, and 7LKRTYQ12 respectively (Fig 3). Preserved identity and location of *rpmH* mutations across *Neisseria* species suggests that this is a conserved mechanism of macrolide resistance across the genus. L34 has been shown be a nonessential ribosomal component in *Bacillus subtilis* [46] and *Escherichia coli* [47]. There is some evidence that L34 may enhance the efficiency of ribosome formation [46,47], however the functional consequences and their relationship to resistance of the amino acid duplications in Nel and Ngo are unclear and will require future investigation. We do not find any of the other Ngo mutations in ribosomal components (C2611T, A2059G, *rplD* G70D, or *rplV* tandem duplications) in Nel. However, we describe novel azithromycin resistance conferring mutations in the intergenic region upstream of *rpsC*, similarly, produced through tandem duplication events (Fig 2). To our knowledge these have not yet been reported in *Neisseria*, and likely impact the expression of *rpsC* due to their location in or proximal to the promoter.

Almost all of the duplications reported here imparted some fitness cost, as measured by *in vitro* growth assays of isogenic cell lines (Fig 4). Ngo duplication mutations in L34 were reported to be transitory stepping stones to acquiring C2611T and A2059G mutations and repeatedly lost in culture [32], further suggesting a fitness cost to *rpmH* duplications in an alternate genetic background. Though most of the *rpsC* mutations also imparted some fitness cost, the duplication in T-LJT-1 appeared to have no impact on fitness (Fig 4), suggesting that it may be sustainable in Nel populations. However, as the nature of *in vitro* growth assays does not replicate the host or the complex microbial community with which bacteria must compete with, these results may not be replicated in natural populations. Interestingly of the 18 Nel genomes reported on NCBI, three had a GTAA duplication present 17 bps upstream of the *rpsC* start site (Fig 2; S1 Table). Though different than the duplications reported here, this suggests the presence of stable genetic variation upstream of *rpsC*. Further work will be needed to elucidate the long-term stability of all uncovered mutations, and to assess if they are either transitory stepping-stones or persistent, when coupled with compensatory mutations (e.g., as is the case for a variant in *acnB* mitigating growth defects in ceftriaxone resistant *penA* mutants [48]), mechanisms of macrolide resistance in commensal *Neisseria* species.

Assessing the likelihood of commensal alleles to be transferred to pathogenic *Neisseria* will aid in determining those resistance mechanisms most at risk of rapid dissemination into pathogen populations, and may guide future prospective genotyping practices during routine public health surveillance efforts and the development of point-of-care resistance diagnostics. Though allelic exchange has been extensively documented across the *Neisseria* [9–18], some barriers to recombination exist such as divergent DNA uptake sequences (DUSs). DUSs are present throughout *Neisseria* genomes and aid in DNA binding to pilus-associated DNA uptake machinery, however slight sequence variations reduce binding efficiency across clades with different DUS types [7]. Though the pathogenic *Neisseria* and Nel harbor different DUSs, the AT-DUS (5’-AT-GCCGTCTGAA-3’) and AG-DUS (5’-AG-GCCGTCTGAA-3’) respectively, recombination has been shown to occur between members of these DUS groups previously [7]. Recent work has also demonstrated that commensal DNA is toxic to Ngo. Since Nel and Ngo have different intrinsic methylases, Ngo restriction enzymes cleave incorporated DNA at heteroduplexes with Nel methylation signatures, resulting in the loss of chromosome integrity and cell death [49,50]. Future work will focus on assessing the (1) possibility of transfer of Nel *rpmH* and *rpsC* alleles into Ngo backgrounds, and (2) quantifying the impact of the above barriers to recombination on genetic exchange between these two species.

Overall, our results expand on prior studies that provide initial insights into illuminating the commensal resistome [27–29], which is a known source of antibiotic resistance for *Neisseria* pathogens [11,13–15,24,25]. We find evidence of constraint on high level macrolide resistance genotypic evolution in AR-0945, with convergence on only two sites in the genome, however different genetic starting places will likely impact evolutionary outcome due to epistatic and additive effects between loci. Thus, future work will not only focus on expanding this approach to other commensal species and antimicrobials, but will incorporate intraspecific variation as an additional consideration. Ultimately, this work emphasizes the power of experimental evolution in characterizing the genetic pathways to resistance in commensal species, which will be key to illuminating mutations at risk of transfer across species boundaries and their effects.

## Methods

### Bacterial strains and growth conditions

The bacterial strain *N*. *elongata* AR Bank #0945 used for this study was obtained from the Centers for Disease Control and Prevention (CDC) and Food and Drug Association’s (FDA) Antibiotic Resistance (AR) Isolate Bank “*Neisseria* species MALDI-TOF Verification panel”. For all experiments, *N*. *elongata* AR Bank #0945 and its evolved derivatives were revived from trypticase soy broth (TSB) stocks containing 50% glycerol stored at -80°C. Stocks were streaked onto GC agar base (Becton Dickinson Co., Franklin Lakes, NJ, USA) media plates containing 1% Kelloggs solution [51] (GCP-K plates), and were grown for 18–24 hours at 37°C in a 5% CO_2_ atmosphere.

Experimental evolution was conducted by passaging 16 replicate stocks of *N*. *elongata* AR Bank #0945 in the presence of azithromycin for 20 days or ~480 generations. A selective gradient of azithromycin was applied to GCB-K plates using Etest strips (bioMérieux, Durham, NC), and each day overnight growth was collected from the entire zone of inhibition (ZOI) and a 1 cm region in the bacterial lawn surrounding the ZOI (Fig 1). Collected cells were suspended in TSB, and plated onto a fresh GCB-K plate with a new Etest strip. MICs each day were determined by reading the lowest concentration that inhibited growth, and reduced susceptibility was determined using the Clinical & Laboratory Standards Institute (CLSI) guidelines for *N*. *gonorrhoeae* (breakpoint AZI ≥ 2 μg/mL) [20]. MICs were read by at least two independent researchers. Five control strains were passaged for 20 days using the same protocol on media containing no selective agent. Final cell populations were streaked onto GCB-K plates and individual colonies were stocked for further analysis.

Final MIC values for post-selection lineages were measured by Etest strips on GCB-K plates, according to the manufacturer specifications–which have been demonstrated to have comparable MIC values to the agar dilution method [52]. In brief, cells from overnight plates were suspended in trypticase soy broth to a 0.5 McFarland standard and inoculated onto a GCB-K plate. Etest strips were subsequently placed on the surface of the plates. Following 18–24 hours of incubation at 37°C in a 5% CO_2_ incubator, MICs were determined and reduced susceptibility was recorded using GISP guidelines. Reported MICs are the mode of 3 independent tests.

### Whole genome sequencing and comparative genomics

Cells from evolved cell lines were lysed by suspending growth from overnight plates in TE buffer (10 mM Tris [pH 8.0], 10 mM EDTA) with 0.5 mg/mL lysozyme and 3 mg/mL proteinase K (Sigma-Aldrich Corp., St. Louis, MO). DNA was purified using the PureLink Genomic DNA Mini kit (Thermo Fisher Corp., Waltham, MA) and treated with RNase A. Sequencing libraries were prepared using the Nextera XT kit (Illumina Corp., San Diego, CA), and uniquely dual-indexed and pooled. Each pool was subsequently sequenced the Illumina MiSeq platform at the Rochester Institute of Technology Genomics Core using V3 600 cycle cartridges (2x300bp).

Sequencing quality of each paired-end read library was assessed using FastQC v0.11.9 [53]. Trimmomatic v0.39 [54] was used to trim adapter sequences, and remove bases with phred quality score < 15 over a 4 bp sliding window. Reads < 36 bp long, or those missing a mate, were also removed from subsequent analysis. The AR-0945 draft assembly was constructed using SPAdes v.3.14.1 [55] and annotated with prokka v.1.14.5 [56]. Trimmed reads were mapped back to the AR-0945 draft assembly using Bowtie2 v.2.2.4 [57] using the “end-to-end” and “very-sensitive” options and Pilon v.1.16 [58] was used to call variant sites. Only single nucleotide polymorphisms and short indels were retained, and any variants called within 100 bp of the end of contigs were removed.

### Transformations

Transformations were conducted by inoculating GCP broth (7.5 g protease peptone 3, 0.5 g soluble starch, 2 g dibasic K2HPO4, 0.5 g monobasic KH2PO4, 2.5 g NaCl, and double-distilled water [ddH2O] to 500 ml; Becton Dickinson Co., Franklin Lakes, NJ) supplemented with 1% IsoVitaleX and 10 mM MgCl (Sigma-Aldrich Corp., St. Louis, MO) with cells to an optical density (OD) of ~0.5. Cell suspensions were subsequently incubated with 100 ng of gDNA for 4 hours to allow DNA uptake, homologous recombination, and expressions of new alleles. Cell suspensions were then serially diluted to allow for quantification of transformation efficiency and spotted onto GCB-K plates containing 4 μg/mL of azithromycin. Cultures then were incubated overnight and after 18 hours colonies were counted for each reaction and azithromycin resistant transformants were selected by picking single colonies. Transformants were subsequently MIC tested and whole-genome sequenced to nominate inherited derived mutations.

Polymerase chain reaction (PCR) and Sanger sequencing were used to confirm the location and identity of all derived polymorphisms nominated in transformants from genomic sequencing screens. In brief, PCR was conducted in 50-μl volumes using Phusion High-Fidelity DNA polymerase (New England Biolabs, Ipswich, MA). Primers for *rpmH* (F: 5’-CGAAGCTTTCCAAAACGGCT-3’; R: 5’-AAGGTTCGGCCAAAGATTGC-3’) and the *rpsC/rplB* intergenic region (F: 5’- ATCGCTACTTTTAGCAAACCACT-3’; R: 5’-TGCAGAGCATAATGAAGGTGCT-3’) were annealed at 60°C. Reactions were conducted for 35 cycles, with 30 second extensions. Resultant products were cleaned using ExoSAP-IT (Applied Biosystems, Foster City, CA), and sequenced via the Sanger method.

### Growth curves

Cells were inoculated into GCP broth supplemented 1% Kelloggs solution to an OD_600_ of ~0.1, using a Genesys 150 spectrophotometer (Thermo Scientific, Waltham, MA). Cell suspensions were distributed into 96-well plates and incubated at 37°C in a BioTek Synergy H1 microplate reader (BioTek, Winooski, VT). Subsequent OD_600_ measurements were taken every hour for 21 hours, with a 1-minute shake at 180cpm prior to reading. The BioTek Gen5 v.3.05 software was used to interpret OD values. Replicates were completed for each cell line (n = 6), and all downstream analyses were performed in R [59].

## Supporting information

S1 Fig Trajectories of macrolide resistance evolution in drug-exposed cell lines. Azithromycin minimum inhibitory concentration values increased from the ancestral AR-0945 stock in all cases, however resistance (≥ 2 μg/mL) emerged in only seven lineages within 20 days.
(PDF)

S1 Table *Neisseria elongata* NCBI deposited genome *rpmH* and *rpsC* variants (n = 18). (XLSX)

S1 File Sanger sequencing files for *rpmH* and *rpsC* for *Neisseria elongata* strains used within this study. (ZIP)
