# Supplementary figures and images for: Evolutionary paths to macrolide resistance in a Neisseria commensal converge on ribosomal genes through short sequence duplications
Source: PLoS One. 2022 Jan 13;17(1):e0262370. doi: 10.1371/journal.pone.0262370 (PMC8758062; doi:10.1371/journal.pone.0262370)

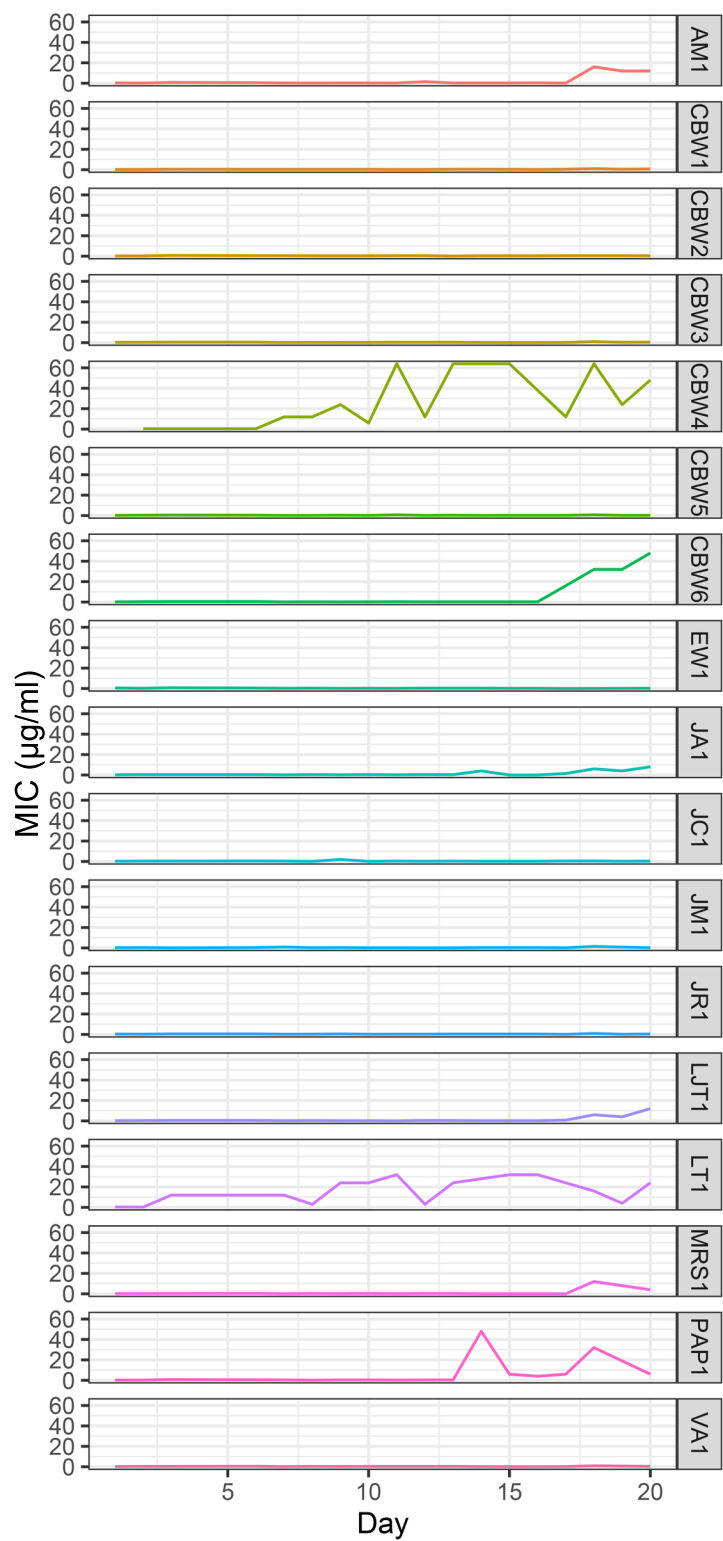

Supplement: S1 Fig — Azithromycin minimum inhibitory concentration values increased from the ancestral AR-0945 stock in all cases, however resistance (≥ 2 μg/mL) emerged in only seven lineages within 20 days. (PDF) [file pone.0262370.s001.pdf]
